# Supplementary material for: An assessment of the quality of antenatal care and pregnancy outcomes in a tertiary hospital in Ghana
Source: PLoS One. 2022 Oct 12;17(10):e0275933. doi: 10.1371/journal.pone.0275933 (PMC9555636; doi:10.1371/journal.pone.0275933)
Supplement: S1 File — (DOCX) [file pone.0275933.s001.docx]

**ANC QUALITY SCORING SYSTEM**

The scoring system for this study combined number of contacts patient made with the health facility, the timing of ANC initiation and the recommended interventions that were actually carried out during those contacts. This scoring system was formulated based on works done by Kyei et al in Africa; Hodgins and D’Agostino and Beeckman et al in Europe [2, 18, and 19].

**Scoring system:**

1. **Number of contacts clients make**: each contact scores 1 point. The maximum point that can be scored is 8 which is equal to the minimum recommended visits by WHO. Making more than 8 contacts does not give additional scoring points.
2. **Booking visit (initiation visit)**:

- Initiating ANC before 14 weeks gives 2 points.
- Initiating ANC late (at 14 weeks and above) scores zero point.

1. **Interventions:**
2. Hemoglobin analysis before third trimester: (maximum of 2 points)

- 2 or more hemoglobin (HB) investigations with normal result equals 2 points
- A single hemoglobin check with normal result scores 1 point
- A low HB without any intervention scores zero point
- A low HB with intervention scores 2 points

1. Syphilis: (maximum of 1 point)

- No screening scores zero
- Negative result after screening scores 1 point
- Screening with positive result and appropriate treatment scores 1 point
- Screening with positive result but no intervention scores zero

1. Hepatitis B: (maximum of 1 point)

- Not screened = 0 point
- Negative result=1 point
- Positive result with intervention=1 point
- Positive result with no intervention= 0 point

1. HIV: (maximum 2 points)

- Not screened= 0 point
- Single screening with negative result=1 point
- Two or more screening with negative results= 2 points
- Positive result with treatment= 2 points
- Positive result without treatment=0 point

1. Ultrasonography: (maximum of 3 scores)

- No sonography during pregnancy= 0 point
- First trimester scan=1 point
- Anomaly scan= 1 point
- Third trimester scan:1 point
- All above three scans done=3 points

1. Intermittent prophylactic treatment: (maximum of 4 points)

- No provision=0 point
- Provision of ITN=1 point
- 4 or more sulfadoxine/pyrimethamine (SP)=2
- Less than 4 SP=1
- Maternal education on malaria=1
- Education on malaria, 4 or more SP plus provision of ITN=4 points

1. Iron and folate supplementation: (maximum of 2 points)

- No supplementation ever in pregnancy= 0 points
- Supplementation from first trimester till delivery=2
- Late supplementation/breaks in intake/ poor compliance=1

h. Tetanus toxoid vaccine: (maximum of 2 points)

- Not given even though required=0 point
- Given but not as recommended (number and timing) =1 point
- Given as recommended (number and timing) =2point

I. Hookworm prophylaxis: (maximum 1 point)

- Given as recommended (after 16 weeks gestation) =1 point
- Never given or not as recommended= 0 point

j. Urinalysis: (maximum 1 point)

- Screening done with normal results=1 point
- No screening done=0 point
- Appropriate treatment/intervention after abnormal result=1 point
- No screening or inappropriate treatment after abnormal results=0 point

k. Blood pressure: (maximum of 4 points)

- Not checked at the ANC: 0 point
- Checked at every contact (with normal levels) = 4 points
- Checked at some contacts (with normal results) =2 points
- Appropriate management of cases=4 points
- No or inappropriate management with elevated levels=0 point

l. Maternal education: (maximum of 4 points)

- No maternal education=0 point
- Mass/group education=1
- Mass plus specific/individualized education=2
- Clients ability to recall 4 or more danger signs in pregnancy after mass and individualized education=4 points

m. Sickle cell screening: (maximum1 point)

- No screening done: 0 point
- Screening done: 1 point
- Appropriate counseling and intervention with positive test result=1 point
- Inappropriate/no counseling with positive test result=0 point

n. Blood group and rhesus determination: (maximum 1 point)

- Not done=0 point
- Testing done: 1 point
- Appropriate counseling after rhesus negative result: 1 point
- No counseling after rhesus negative: 0 point

o. Stool analysis: (maximum score point 1)

- Analysis not carried out=0 point
- Analysis done with normal results=1 point
- Appropriate treatment with abnormal result=1 point
- No/inappropriate treatment with abnormal results=0

**Table 1. Frequency distribution of interventional scores (N=950)**

| **Interventional scores** | **Frequency** | **Percentage (%)** |
| --- | --- | --- |
| Haemoglobin |  |  |
| 0 | 88 | 9.26% |
| 1 | 562 | 59.16 |
| 2 | 300 | 31.58 |
| Syphilis |  |  |
| 0 | 205 | 21.58 |
| 1 | 745 | 78.42 |
| Hepatitis B |  |  |
| 0 | 271 | 28.53 |
| 1 | 679 | 71.47 |
| HIV |  |  |
| 0 | 191 | 20.11 |
| 1 | 512 | 53.89 |
| 2 | 247 | 26.0 |
| Ultrasonography |  |  |
| 0 | 96 | 10.11 |
| 1 | 410 | 43.16 |
| 2 | 235 | 24.74 |
| 3 | 209 | 22.0 |
| Malaria prevention |  |  |
| 1 | 950 | 100 |
| 2 | 950 | 100 |
| 3 | 750 | 78.94 |
| 4 | 56 | 5.89 |
| Iron and folate supplementation |  |  |
| 0 | 25 | 2.63 |
| 1 | 471 | 49.58 |
| 2 | 454 | 47.80 |
| Tetanus toxoid vaccine |  |  |
| 0 | 56 | 5.89 |
| 1 | 545 | 57.37 |
| 2 | 349 | 36.74 |
| Hookworm prophylaxis |  |  |
| 0 | 796 | 83.79 |
| 1 | 154 | 16.21 |
| Urinalysis |  |  |
| 0 | 399 | 42.0 |
| 1 | 551 | 58.0 |
| Blood pressure |  |  |
| 0 | 5 | 0.53 |
| 2 | 25 | 2.63 |
| 4 | 920 | 96.84 |
| Maternal education |  |  |
| 0 | 18 | 1.89 |
| 1 | 907 | 95.47 |
| 2 | 350 | 36.84 |
| 4 | 295 | 31.05 |
| Sickling screening |  |  |
| 0 | 233 | 24.53 |
| 1 | 717 | 75.47 |
| Blood group and rhesus determination |  |  |
| 0 | 118 | 12.42 |
| 1 | 832 | 87.58 |
| Stool analysis |  |  |
| 0 | 835 | 87.89 |
| 1 | 115 | 12.11 |
