# Supplementary material for: An assessment of the quality of antenatal care and pregnancy outcomes in a tertiary hospital in Ghana
Source: PLoS One. 2022 Oct 12;17(10):e0275933. doi: 10.1371/journal.pone.0275933 (PMC9555636; doi:10.1371/journal.pone.0275933)
Supplement: Attachment — Submitted filename: PLOS ONE article reviewed COMMENTS ON ARTICLE.docx [file pone.0275933.s003.docx]

**COMMENTS ON ARTICLE**

**TITLE:** The title predicts and reflects the type and tone of the study. It has no redundant words that should be removed. However, the key words are too many and should be reduced to six words.

**ABSTRACT:** This is structured, well written and appropriate in length.

**INTRODUCTION:** This contains a brief background information on the subject of study. It provides explanation as to why the subject is important. The research questions, objectives and justification are well defined. Literature review is not critical enough but some gaps in existing literature were identified.

**METHODOLOGY:** The study setting was well described with relevant information and indices. The design was reproducible. The eligibility criteria were well defined and the research objectives were met. The research tools were well described. Ethical approval for the research was obtained. The sample size determination formula, process, assumptions were clearly documented and appropriate. Appropriate study tools were described and attached as appendices. Statistical analysis was appropriately described.

**RESULTS:** The results were in relevant graphics and tables.

Table 1 (Attachment): it is not clear if this table reflects the total scores or number of participants as it concerns malaria prevention and maternal education; the sum of the figures in these columns are more than 950 ( the total number of participants). This will need to be reviewed as the criteria for scoring are clearly stated and no single person can have more that one score.

**DISCUSSION:** This section is well written. However, the authors did not emphasize the importance of malaria infection as a cause of anaemia in pregnancy in the tropics. The author in concluding advised the Government to ensure that pregnant women receive high quality ANC through through health education and care but did not mention the training and retraining of health workers, and improvement of health facilities

**THIS ARTICLE IS ACCEPTABLE FOR PUBLICATION AFTER TABLE 1 HAS BEEN REVIEWED.**
